# Effects of COVID-19 Prevention Measures on Other Common Infections, Taiwan

**DOI:** 10.3201/eid2610.203193

**Published:** 2020-10

**Authors:** Hong-Hsi Lee, Sheng-Hsuan Lin

**Affiliations:** New York University School of Medicine, New York, New York, USA (H.H. Lee);; National Chiao Tung University, Hsinchu, Taiwan (S.H. Lin)

**Keywords:** 2019 novel coronavirus disease, coronavirus disease, COVID-19, severe acute respiratory syndrome coronavirus 2, SARS-CoV-2, viruses, respiratory infections, zoonoses, influenza, enterovirus, scarlet fever, Taiwan

## Abstract

To determine whether policies to limit transmission of severe acute respiratory syndrome coronavirus 2 (SARS-CoV-2) hinder spread of other infectious diseases, we analyzed the National Health Insurance database in Taiwan. Rates of other infections were significantly lower after SARS-CoV-2 prevention measures were announced. This finding can be applied to cost-effectiveness of SARS-CoV-2 prevention.

Drawing from experience with the severe acute respiratory syndrome epidemic in 2003, the Taiwan government has established a central command system for a quick response to epidemics arising from China (*1*). Since the first confirmed case of coronavirus disease (COVID-19) in Taiwan was reported, Taiwan officials acted immediately with regard to border control, public health education (mask wearing and handwashing), ensuring adequate medical equipment, and early suspension of classes. These policies may not only reduce the spread of severe acute respiratory syndrome coronavirus 2 (SARS-CoV-2) but may also have similar effects on spread of other infectious diseases (*2*,*3*). Using nationwide weekly surveillance data (*4*), we compared the activity of common infections during 2015–2020 with the timeline of actions and policies implemented to protect against spread of SARS-CoV-2 in Taiwan.

The Taiwan National infectious Disease Statistics System (*4*) from the Taiwan Centers for Disease Control monitors emergency and outpatient visits for patients with acute infections, diagnosed according to clinical manifestations and laboratory results in 181 hospitals (covering 97.5% of emergency visits), through the National Health Insurance database and reports weekly statistical data. Using these data, we compared the number of outpatient visits for influenza, pneumonia, enterovirus infection, and scarlet fever and the number of confirmed cases of severe complicated influenza in the 2019–20 influenza season (week 40 in 2019 through week 18 in 2020; 1,931,471 cases) versus the same data for the 5 previous influenza seasons (10,688,851 total cases).

To estimate the change in outpatient visits or confirmed case numbers (hereafter called activity) after the COVID-19 outbreak (weeks 1–18 in 2020), we used a difference-in-difference regression model. The model included a categorical variable for each week, a categorical variable for each year, and the interaction variables for each week after the outbreak and for the 2019–20 season. Because of concerns about the COVID-19 pandemic, during the first quarter of 2020, the overall number of hospital visits in Taiwan dropped by 14%. We conducted a sensitivity analysis by multiplying 1/(1 – 0.14) times the number of cases for the 5 selected diseases during these periods. Institutional board review was not required because we used only deidentified, secondary statistical data for this study.

Overall infection activity was lower during the 2019–20 season than during the 5 previous seasons. For the 2019–20 season, activities of all 5 diseases notably decreased after weeks 6–7 (Figure). According to the difference-in-difference analysis, activities of influenza and severe complicated influenza were significantly lower after week 7 during the 2019–20 season than during the 5 previous seasons. Comparing the 2019–20 season with the 5 previous seasons, outpatient pneumonia activity was lower after week 8, enterovirus activity after week 16, and scarlet fever activity after week 10 (Table; Figure).

**Figure:**
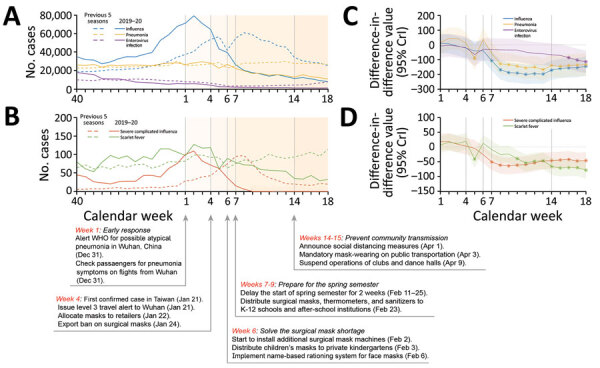
Infection activities and measures against coronavirus disease (COVID-19) in Taiwan, 2015–2020, comparing activities of influenza, severe complicated influenza, pneumonia, enterovirus infection, and scarlet fever during the 2019–20 influenza season versus the same data for the 5 previous influenza seasons by using difference-in-difference analysis. A) Number of cases of influenza, pneumonia, and enterovirus infection; B) number of cases of severe complicated influenza and scarlet fever; C) difference-in-difference value in 2020 vs. 2015–2019 (95% credible interval [CrI]) for influenza, pneumonia, and enterovirus infection; D) difference-in-difference value in 2020 vs. 2015–2019 (95% CrI) for severe complicated influenza and scarlet fever. Negative 95% CrI indicates fewer cases in the 2019–20 season than in the 5 previous seasons (p<0.05). Vertical dotted lines indicate timeline of actions and policies against COVID-19 (weeks 4, 6, 7, and 14; see panel B). WHO, World Health Organization; K-12, kindergarten through 12th grade.

**Table:** Statistical significance according to difference-in-difference analysis of activities of influenza, severe complicated influenza, pneumonia, enterovirus infection, and scarlet fever during the 2019–20 season versus 5 previous seasons, Taiwan, 2015–2020*

Calendar week	p value				
	Influenza	Severe complicated influenza	Pneumonia	Enterovirus	Scarlet fever
1	0.76	0.22	0.71	0.83	0.64
2	0.83	0.21	0.26	0.94	0.27
3	0.80	0.53	0.27	0.81	0.41
4	0.52	0.82	0.76	0.62	0.19
5	0.22	0.75	**0.02**	0.10	**0.005**
6	0.25	0.08	0.33	0.44	0.38
7	**0.002**	**<0.001**	0.13	0.46	0.56
8	**<0.001**	**<0.001**	**0.001**	0.38	0.09
9	**<0.001**	**<0.001**	**<0.001**	0.37	0.11
10	**<0.001**	**<0.001**	**<0.001**	0.37	**0.008**
11	**<0.001**	**<0.001**	**<0.001**	0.21	**<0.001**
12	**<0.001**	**<0.001**	**<0.001**	0.15	**0.001**
13	**<0.001**	**0.001**	**<0.001**	0.13	**0.002**
14	**<0.001**	**0.002**	**<0.001**	0.09	**<0.001**
15	**<0.001**	**0.003**	**<0.001**	0.07	**<0.001**
16	**<0.001**	**0.004**	**<0.001**	**0.03**	**<0.001**
17	**<0.001**	**0.001**	**<0.001**	**0.009**	**<0.001**
18	**<0.001**	**0.002**	**<0.001**	**0.004**	**<0.001**

*Boldface indicates significance at p<0.05.

In Taiwan, infection rates for 5 selected diseases were lower in 2020 than in previous seasons. This observation correlates with implementation of actions and policies against COVID-19, such as early vigilance and taking proactive measures to prevent droplet and contact transmission in public and at schools. The effect of social distancing in Taiwan was unclear because related measures were not officially announced until the COVID-19 pandemic started subsiding in early April (*4*). These policies potentially have indirect effects on noninfectious diseases associated with acute viral infections, such as myocardial infarction and ischemic stroke (*5*,*6*). By comparing the cost of SARS-CoV-2 prevention and the effect on the economy and health during the pandemic in Taiwan and other areas, we could evaluate the cost-effectiveness of these measures and use this information to develop policies for future disease control.
